# Supplementary material for: Learning curve and surgical outcome of robotic assisted colorectal surgery with ERAS program
Source: Sci Rep. 2022 Nov 29;12:20566. doi: 10.1038/s41598-022-24665-w (PMC9709162; doi:10.1038/s41598-022-24665-w)
Supplement: Supplementary file 1 — Supplementary Information 1. [file 41598_2022_24665_MOESM1_ESM.zip › LSC Raw data-Ñ[▒K/084/H_ñw╝╨░O▒Kñσ.pdf]

| | | |
|---|---|---|
| 正式摘要(電子簽章：000000195268257)<br>W52 / W52 ☐英文標題(注意:無英文姓名-請補病人基本檔!) | ☐【讀取】憑證 | |

# 臺中榮民總醫院
## 出院病歷摘要

轉入號： 轉出號：

[redacted]

| | |
|---|---|
| (8)流水編號 | |
| (9)入院日期 | 109/07/14 (2020) 直腸外科 W52 - 161 |
| (10)轉科日期 | |
| (11)出院日期 | 109/07/23 (2020) 住院天數計 9 日 |

## 診斷

- 入院 :adenocarcinoma of sigmoid colon
- 出院 :Adenocarcinoma of sigmoid colon s/p laparoscopic anterior resection and anastomosis on 2020/7/16.

## 主訴

- bloody stool for 5 months

## 病史

- This 85 years old female has past history of hypertension. She had an episode of bloody stool since this Febuary. There was no changed bowel habbit, decreased appetite or obvious body weight loss. She came to our OPD for help and colonoscopy was done on 6/15 that a colon tumor at 45cm from anal verge was seen. Pathology report showed moderately differentiated adenocarcinoma. Further survey didn't showed metastasis. Thus, she was admitted for operation.

## 體檢發現

- Anal inspection:
  Recto digital examination:
  Sigmoidoscopy:
  Anoscopy:
  Abdomen:
  - General appearance :
    - easy looking , conscious clear , without cardiopulmonary distress
    - Vital signs自動帶入date 體溫(BT) 脈搏(P) 呼吸(R) 血壓(BP) 身高(BH) 體重(BW) BMI
      20200714 35.7℃ 75次/分 18次/分 159/97mmHg 151.7cm 66.5kg 28.9
    - Vital signs: B.P. 159/97 mmHg , P.R. 75 /min , R.R. 18 /min , B.T. 35.7 ℃
  - HEENT :
    - Eyes: no anemic conjunctiva, no icteric sclera, normal EOM
    - ENT: no otorrhea, no rhinorrhea, no erythematous mucosa, no injected tonsil, no deformity
  - Neck: supple, freely movable, trachea midline,no deformity, no engorged jugular vein , no palpable thyroid or lymph node enlargement
  - Chest:
    - Inspection: Grossly normal, symmetric and fully expansion, no accessory muscle used, no operation scar, no spider angiomata, no gynecomastia
    - Palpation: no palpable mass or nodule, no subcutaneous emphysema, no tenderness
    - Ausculation: clear breathing sound
  - Heart:
    - Palpation: no thrill, no heave
    - Percussion: normal heart size
    - Ausculation: regular heart beat, normal S1, S2, no gallop, no murmur
  - Extremities: No deformity, full ranged of motion without limitation, no clubbing finger or nail cyanosis, no pitting edema, no ulcer or pigmentation, no obvious skin lesion, no varicose vein, no bone, joint or muscle tenderness

### CRS CONDITION

- Karnofsky performance status: (90) Able to carry on normal activity; minor signs or

- symptoms of disease
- PE of Abdomen:
- __1. Inspection: flat
- __2. Ausculation: normoactive
- __3. Palapation: soft
- __4. Percussion: -
- Digital Rectal Examination: less bloody stool
- CEA: 34.32 ng/ml
- Imaging Studies:
- Colonoscope report: 1.Colon tumor2.Colon polyp
- Sono , CT of abdomen and chest Films:1. Mild wall thickening of colon at junction of D- and S- colon around the clips, suggest correlate with findings of colonoscopy.
  2. Two GGNs at RUL and lingula of LUL, suggest further evaluation or close follow-up.
- Figures:
  1. colon & rectum

## 手術日期及方法

- 手術口期及方法20200716 Laparoscopic Anterior resection and anastomosis - malignant

## 住院治療經過

- After admission, operation of laparoscopic anterior resection and anastomosis was done on 2020/7/16. We also consulted REHA, PAIN, and CCRT for facilitating post-op recovery according to ERAS protocol. Flatus and stool passage noted on POD1 with only mild pain under epidural PCA use. We removed Foley on POD1 but failed due to poor self-urination status. So we tried again on next day(POD2) and added bethanechol, normal self-urination noted after Foley removal. With decreasing penrose drainage amount and increasing appetite, we removed CVP and penrose drain ON 7/22. Under relatively stable condition, she is allowed to be discharged today.

## 合併症

- nil

## 一般檢查

- 累積報告 :

| DATE | TIME | ALB | TP | BIL.T | BIL.D | ALKP | AST | ALT | LDH | CHOL | TG | HDL-C | LDL | UA | NH3 | AMY |
|---|---|---|---|---|---|---|---|---|---|---|---|---|---|---|---|---|
| 1090714 | 2158 | 3.8 | 6.6 | 0.4 | 0.1 | 108 | 33 | 18 | 278 | | | | | | | |
| 1090716 | 1513 | 3.6 | | 0.4 | 0.1 | 102 | 29 | 17 | | | | | | | | |
| 1090720 | 0936 | | | | | | 22 | 13 | | | | | | | | |
| 1090722 | 1041 | | | | | | 28 | 15 | | | | | | | | |

| DATE | TIME | WBC | RBC | HGB | HCT | MCV | PLT | BLST | NEUT | BAND | SEG | LYM | MON | EOS | BAS |
|---|---|---|---|---|---|---|---|---|---|---|---|---|---|---|---|
| 1090714 | 2155 | 6050 | 4.16 | 11.4 | 37.1 | 89.2 | 269 | | 60.0 | | | 31.4 | 6.0 | 2.3 | 0.3 |
| 1090716 | 1509 | 11120 | 4.30 | 11.9 | 37.3 | 86.7 | 271 | | 91.7 | | | 7.3 | 0.9 | 0.0 | 0.1 |
| 1090720 | 0857 | 6940 | | 11.3 | 35.6 | | 205 | | 69.7 | | | 18.4 | 5.5 | 6.3 | 0.1 |
| 1090722 | 1042 | 5820 | 3.62 | 10.3 | 31.8 | 87.8 | 236 | | 73.4 | | | 14.9 | 6.2 | 5.3 | 0.2 |

| DATE | TIME | NA | K | CL | CA | BUN | CREAT | Mg | P | CRP | ESR | PCT | LIPASE | LACTATE |
|---|---|---|---|---|---|---|---|---|---|---|---|---|---|---|
| 1090714 | 2158 | 137 | 4.3 | 106 | 8.7 | 17 | 1.43 | | | | | | | |
| 1090716 | 1513 | 137 | 3.7 | 105 | 8.9 | 8 | 1.04 | | | | | | | |
| 1090720 | 0936 | 138 | 4.3 | | 8.0 | 12 | 1.02 | | | | | | | |
| 1090722 | 1041 | 137 | 4.2 | | 8.1 | 18 | 1.07 | | | | | | | |

| DATE | TIME | GLUCOSE | PAB | TRF | ZN | CU | KETONE | CK | CKMB | PT-P | PT-C | APTT-P | APTT-C | TRO-T |
|---|---|---|---|---|---|---|---|---|---|---|---|---|---|---|
| 1090714 | 2154 | | | | | | | | | 10.3 | 10.3 | 25.4 | | |
| 1090716 | 1511 | 161 | | | | | | | | | | | | |

| DATE | TIME | SP-GR | PH | PROT | GLUC | KETON | BILI | UROB | OB | NITR | LEU | RBC | WBC | EPCEL |
|---|---|---|---|---|---|---|---|---|---|---|---|---|---|---|
| 1090722 | 1210 | 1.009 | 6.0 | Negative | Negative | Negative | Negative | 0.2 | Negative | Negative | 3+ | 2-5 | + | 10-20 |

- 放射線報告 :**檢驗項目 :CHEST PA VIEW 報告日期 :1090715**

  主　訴：
  Result:
  Finding:
  1. Tortuous, calcified and dilatation of thoracic aorta.
  2. Slight enlargement of cardiac shadow.
  3. Slight increase infiltration of bil. lungs.
  4. Scalloping of right hemidiaphragm.
  5. DJD change and scoliosis of T and L spine.
  Impression:
  As above description.

## 特殊檢查

## 病理報告

- 活體組織切片病理報告　手術日期：**109/06/15**　病理號：**S0916912**

  診斷醫師:陳律臻 報告日期:109/06/18
  Pathologic diagnosis:
  1. Colon, 45 cm from anal verge, "A", endoscopic biopsy ---
  Adenocarcinoma, moderately differentiated.
  2. Rectum, 10 cm from anal verge, "B", endoscopic biopsy ---
  Tubulovillous adenoma.
  Ancillary study for diagnosis:
  Recut and AE1/AE3 IHC stain done on section A.
  Prognostic and predictive factor:
  1. Degree of dysplasia: Low grade in B.
  2. Margin of stalk: Cannot be well evaluated in B.
  Gross description:
  The specimen consists of 1) a piece of tan soft tissue, 0.2x0.2x0.2 cm, labeled as "colon, 45 cm". 2) 3 pieces of tan soft tissue, up to 1.1x0.7x0.6 cm, labeled as "rectum, 10 cm". All for section: A) specimen A B) specimen B.
  Microscopic description:
  Section A shows colon mucosa with moderately differentiated adenocarcinoma. Section B shows colon mucosa with tubulovillous glands proliferation with dysplasia.
  Note:
  The diagnosis is concurred in intradepartment consensus meeting on 2020/06/18.
  #T-59300_2 #M-81403_2 2 1340 00CM0F
  #T-59300_2 #M-82630_2 0 1340 000000

## 其它

## 出院時情況

- 3.改門診治療

## 出院指示

| 藥名 | 劑量 | 劑量單位 | 用法 | 頻次 | 數量 | 數量單位 | 現況 | 天數 | 單位 |
|---|---|---|---|---|---|---|---|---|---|
| Ultracet tab | 1 | TAB | PO | Q6HPRN | 12 | TAB | TH | 3 | 天 |
| Norvasc tab 5mg | 1 | TAB | PO | QD | 5 | TAB | TH | 5 | 天 |
| Mag. Oxide tab | 1 | TAB | PO | TID | 15 | TAB | TH | 5 | 天 |

## 出院計劃

- 門診日期
  - date on 2020/07/28
- 門診追蹤

主治醫師 2062F 蔣鋒帆　/　住院醫師 9889F 陳俊男

正式摘要(**電子簽章：000000195268257)**

W52 / W52

doctorRank:B
